# Scallion White and Ginger Extracts Alleviate Stress-Induced Muscle Quality Deterioration in Crucian Carp During Transportation

**DOI:** 10.3390/foods15101645

**Published:** 2026-05-08

**Authors:** Ling Peng, Liangzi Zhang, Chaoping Liu, Tao Yin, Juan You, Ru Liu, Dan Jia

**Affiliations:** 1 College of Animal Science and Technology, Yunnan Agricultural University, Kunming 650201, China; 2 College of Food Science and Technology, Wuhan Business University, Wuhan 430056, China; zlzwbu@163.com; 3 College of Food Science and Technology, Huazhong Agricultural University, Wuhan 430070, China; pengling@webmail.hzau.edu.cn (L.P.); liuchaoping2025@163.com (C.L.); juanyou@mail.hzau.edu.cn (J.Y.); liuru@mail.hzau.edu.cn (R.L.)

**Keywords:** ginger extract, scallion white extract, stress response, antioxidant, muscle quality, gene expression, 10 °C transportation

## Abstract

This study evaluated the effects of scallion white and ginger extracts on stress indicators, gene expression, tissue structure, and muscle quality in crucian carp during transportation. Compared with the control group, ginger extract effectively alleviated long-term transportation stress (48 h), as evidenced by lower levels of glucose and lactate dehydrogenase activity, along with reduced pathological damage in gill, liver, and muscle tissues. Consequently, muscle quality parameters including shear force, glycogen content, and inosine monophosphate levels were notably improved. These improvements were associated with the suppression of heat shock responses, inflammation, and apoptosis, supported by the downregulation of *hsp70*, *il-6*, *caspase 3*, *caspase 8* and *bax* gene expression. Similar trends were observed in the scallion white extract group, though its anti-stress effects and muscle quality enhancement were comparatively weaker. The findings suggest that natural extracts offer a promising approach to mitigating stress and improving muscle quality during live fish transportation.

## 1. Introduction

With the rapid growth of e-commerce logistics, single-package live fish transportation has become a key approach for delivering fresh aquatic products directly from farms to consumers. However, long-distance transport often leads to stress-induced declines in survival and muscle quality due to environmental fluctuations such as oxygen depletion, vibration, and temperature changes [1]. Conventional stress-mitigation strategies (e.g., physical cooling, fasting, anesthetics) show limited efficacy and raise concerns about complexity and drug residues, underscoring the need for safe natural alternatives [2,3,4].

Scallion white (*Allium fistulosum*) and ginger (*Zingiber officinale*) contain abundant bioactive compounds with antioxidant, anti-inflammatory, and antimicrobial activities [5,6]. Previous studies have shown that their extracts can scavenge reactive oxygen species, regulate inflammatory pathways, and even exert anesthetic-like effects, though their application in live fish transport remains unexplored. Wang et al. [7] demonstrated that scallion white extract protects cellular membranes by scavenging reactive oxygen species (ROS) and inhibiting the NF-κB signaling pathway, thereby reducing the release of pro-inflammatory mediators. Fatemi et al. [8] and Meng and Sun [9] further reported that ginger extract can act as both an antioxidant and antibacterial agent in meat and oil preservation by eliminating free radicals and delaying lipid oxidation. Additionally, gingerols have been shown to exert anesthetic-like effects by suppressing central nervous system excitability and reducing metabolic intensity, while enhancing the activity of endogenous antioxidant enzymes such as SOD and glutathione peroxidase (GPx). The extracts of ginger and scallion whites boast excellent raw material accessibility and cost-effectiveness. Although previous studies have mainly investigated the antioxidant, anti-inflammatory, and antimicrobial properties of scallion white and ginger extracts in food preservation, their application in live fish transportation—especially regarding survival and stress responses during single-package oxygenated transport—remains largely unexplored.

This study aimed to address the limited understanding of the effects of commonly used natural antioxidant extracts on physiological stress responses and muscle quality in live fish during transportation. Specifically, the effects of ginger and scallion white extracts on crucian carp were investigated by assessing hematological stress indicators, tissue morphology, gene expression, and muscle quality. By comprehensively evaluating systemic stress responses and post-transport muscle quality, this study highlights the practical potential of scallion white and ginger extracts as natural anti-stress additives in live fish transportation.

## 2. Materials and Methods

### 2.1. Animal Materials

Crucian carp used in this experiment were obtained from the Wuhan Smart Agricultural Research Aquatic Products Base, with an average body weight of 220 ± 30 g and a body length of 23 ± 3 cm. Live fish transport bags were purchased from Xinsu Plastic Products Factory, Baoding, China (20 cm × 30 cm; thickness: 18 strands; material: PE + PET). Ginger extract and scallion white extract were supplied by Shanghai Mingda Biotechnology Co., Ltd., Shanghai, China.

### 2.2. Ethics Statement

All animal experiments were approved by the Animal Care and Use Committee of Huazhong Agricultural University (HZAUF-2024-0042).

### 2.3. Experimental Design

Crucian carp were harvested from the ponds of the Wuhan Smart Agricultural Research Aquatic Products Base and transported to the laboratory within 30 min under oxygenated conditions. Upon arrival, the fish were transferred into aerated tap water for at least 2 days. To eliminate pathogens, fish were immersed in a potassium permanganate solution for 15 min, and individuals exhibiting low vitality (e.g., side-lying behavior) were removed. Healthy and active fish were then placed in rearing tanks equipped with an aeration pump, filter cotton, bacterial house, and ultraviolet lamp, and acclimated without feeding for 2 d.

Following acclimation, 45 fish of uniform size and good vitality were selected for single-package transportation experiments. The fish were randomly divided into three groups: control group, scallion extract group, and ginger extract group. All three groups are subjected to the same simulated transportation operation. The specific packaging process is as follows: the fish-to-water ratio was maintained at 1:3, with aerated tap water as the water source. Packaging was performed as follows: each transport bag (1 fish/bag) was filled with aerated tap water or solution containing the designated extract, the air was expelled, and the bag was sealed using a thermoplastic sealer. Oxygen was then injected until the internal pressure reached approximately 12 kPa. The sealed bags were placed in insulated foam boxes (290 × 170 × 190 mm) with ice packs equivalent to 50% of the total fish and water weight, and subjected to simulated transportation on a vibration platform (120 r/min), with the temperature maintained at 10 °C. The point of transportation time reached 0, 12, 24, 36, 48 h. The scallion white group was supplemented with scallion white extract (0.1 mg/mL) in water, and the ginger group was supplemented with ginger extract (0.1 mg/mL) in water. The concentration used in this study was selected based on the results of our previous research.

#### Sampling

Prior to blood sample collection, MS-222 solution (120 mg/L) was injected into the fish bags using a syringe to minimize the stress response in fish. Once the fish reached a deep anesthesia stage, they were removed from the bags for sample collection.

Blood sample: Blood samples were collected via the caudal vein. A 2 mL syringe was inserted at an angle into the vessel beneath the vertebrae at the tail, and blood was quickly drawn and transferred into a 1.5 mL centrifuge tube. Whole blood samples were temporarily stored in 2 mL centrifuge tubes. After resting at 4 °C for 1 h, samples were centrifuged at 1699× *g* for 10 min at 4 °C. The supernatant was collected into cryovials, rapidly frozen in liquid nitrogen, and then transferred to a −80 °C freezer for long-term storage.

Muscle sample: After descaling, beheading, and evisceration, the fish were fileted along the backbone, and dorsal muscle samples were collected from the region below the dorsal fin and above the lateral line. Some fresh samples were used for immediate measurement of color, shear force, pH, and water-holding capacity. The remaining samples were portioned into self-sealing bags, snap-frozen in liquid nitrogen within 0.5 h after fish death, and stored at −80 °C until further analysis.

### 2.4. Survival Rate

The survival rate of fish was calculated according to the method described by Peng et al. [10].

### 2.5. Water Quality Parameters

pH was determined with a pH meter (PH818, Dongguan, China); dissolved oxygen was measured using a dissolved oxygen meter (AR8210, Dongguan, China); salinity was measured with a salinity meter (AZ8373, Shenzhen, China); and ammonia nitrogen was determined using an ammonia nitrogen analyzer (YC7200-N, Shenzhen, China).

### 2.6. Blood Stress Indicators

Cortisol (COR) levels were measured using a commercial enzyme-linked immunosorbent assay (ELISA) kit following the instructions of manufacturer. Lactic dehydrogenase (LDH), cholesterol (CHOL), triglycerols (TG), glucose (GLU), aspartate aminotransferase (AST), urea (UREA) and creatinine (CREA) were determined using an automated biochemical analyzer according to standard procedures.

### 2.7. Redox System Indicators

Glutathione peroxidase (GPX), catalase (CAT), malondialdehyde (MDA), and superoxide dismutase (SOD) activities were measured using commercial assay kits according to the instruction of manufacturer.

### 2.8. Gill and Liver Tissue Structure

The observation of gill and liver tissue structures was performed as described by Peng et al. [11].

### 2.9. Genge Expression in Blood

The expression levels of *hsp70*, *hsp90*, *caspase 3*, *il-6* gene were determined by RNA extraction, reverse transcription, and quantitative real-time PCR (qRT-PCR) following the methods described by Peng et al. [10]. Primer sequences are listed in Table S1.

### 2.10. Muscle Quality

Shear force: Texture was measured using a texture analyzer (HDP-BS probe) at 1 mm/s and 100% compression, with maximum force (kg) recorded in “Hold” mode.

pH: Two grams of fish muscle were homogenized with 20 mL distilled water, centrifuged at 2000× *g* for 10 min at 4 °C, and the pH of the supernatant was measured.

Drip loss: Dorsal muscle samples were cut into small pieces, weighed, and suspended at 4 °C. After 24 h, the samples were reweighed, and drip loss was expressed as the percentage of weight lost relative to the initial weight.

Color: Muscle samples were measured with a handheld colorimeter, and whiteness was calculated as described by Peng et al. [10].

Muscle glycogen and lactic acid: Muscle glycogen and lactic acid contents were determined using commercial assay kits according to the instruction of manufacturer.

ATP and its metabolites: The sample preparation and high-performance liquid chromatography (HPLC) procedures were performed following the methods described by Peng et al. [10].

### 2.11. Gene Expression in Fish Muscle

The expression levels of *caspase 3*, *caspase 8*, *caspase 9*, *bcl-2*, and *bax* genes were determined by RNA extraction, reverse transcription, and quantitative real-time PCR (qRT-PCR) following the methods described by Peng et al. [11]. Primer sequences are listed in Table S2.

### 2.12. Cellular Structure

Samples were fixed, processed, and sectioned according to the method of Peng et al. [11]. Sections were then examined under a light microscope to assess cellular morphology and structural integrity.

### 2.13. Data Statistical Analysis

All measurements were performed in at least triplicate, and the mean ± standard values were used for statistical analysis. Data were analyzed using SPSS 27.0 (IBM Corp., Armonk, NY, USA) with one-way ANOVA and polynomial contrasts. Post hoc comparisons were conducted using LSD and Duncan tests (*p* < 0.05). Homogeneity of variance was checked, and graphs were generated using GraphPad Prism 9 (GraphPad Software, San Diego, CA, USA).

## 3. Results

### 3.1. Changes in Water Quality Parameters During Transportation

Changes in water quality during transportation are shown in Figure S1. Water pH gradually decreased from slightly alkaline to slightly acidic with increasing transportation time, reaching the lowest level at 48 h; the ginger extract group maintained a higher pH than the control. Dissolved oxygen (DO) levels first increased and then declined, peaking at 24 h, with no significant differences among groups throughout transportation (*p* > 0.05). Ammonia nitrogen and total dissolved solids (TDS) concentrations increased continuously over time; during 24–48 h, both ginger and scallion white groups showed significantly lower ammonia nitrogen and TDS levels than the control (*p* < 0.05), with the lowest values observed in the ginger group at 48 h. Nitrite concentration also increased with transportation time, and during 12–48 h, the ginger extract group exhibited significantly higher nitrite levels than the other groups (*p* < 0.05).

### 3.2. Changes in Blood Stress Indicators of Fish During Transportation

**Figure 1:**
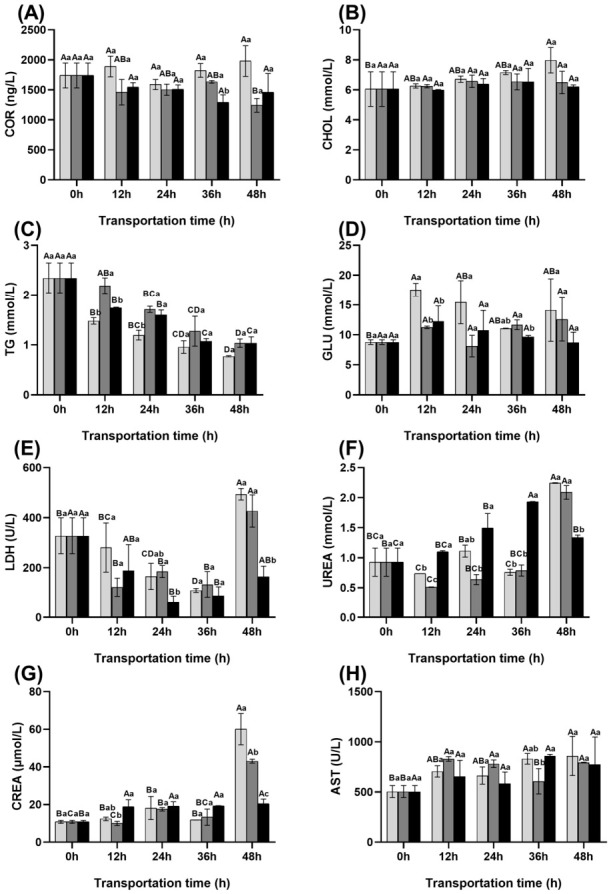
Changes in blood stress indicators of fish during transportation. (**A**) COR; (**B**) CHOL; (**C**) TG; (**D**) GLU; (**E**) LDH; (**F**) UREA; (**G**) CREA; (**H**) AST. 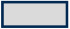 Control group 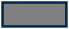 Scallion white group 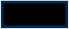 Ginger extract group. COR: Cortisol, CHOL: Cholesterol, TG: Triglycerols, GLU: Glucose, LDH: Lactic dehydrogenase, UREA: Urea, CREA: Creatinine, AST: Aspartate aminotransferase. Different uppercase letters indicate significant differences within the group at different time points (*p* < 0.05), while different lowercase letters indicate significant differences between groups at the same time point (*p* < 0.05).

The changes in physiological parameters during transportation are shown in Figure 1.

Cortisol concentrations remained similar in the control and ginger extract groups throughout transportation (*p* < 0.05). In contrast, the scallion extract group exhibited a significant decrease after 48 h, from 1740 ng/L to 1243.13 ng/L, compared with the control group (*p* < 0.05). With the prolongation of transportation time, CHOL in the control group gradually increased and peaked at 48 h, whereas CHOL levels in the scallion white and ginger extract groups remained relatively stable (Figure 1). TG concentrations decreased significantly over time, but were consistently higher in the ginger extract group and intermediate in the scallion white group compared with the control group. GLU peaked at 12 h in both the control and ginger extract groups, while LDH exhibited a decreasing–increasing trend during transportation. At 48 h, LDH levels were markedly lower in the ginger extract group than in the control and scallion white groups (*p* < 0.05). With prolonged transportation, UREA showed fluctuation patterns in the ginger and scallion white extract groups, while CREA increased steadily in all groups. At 48 h, both UREA and CREA peaked in the control and scallion white groups but remained markedly lower in the ginger group (Figure 1F,G). AST generally increased over time, with lower levels in the ginger group throughout transport. In the scallion white group, AST was higher than in the ginger group and control at 12–24 h, but fell below the control by 36–48 h (Figure 1H).

### 3.3. Changes in Gill and Liver Tissue Structure of Fish After 48 h of Transportation

**Figure 2:**
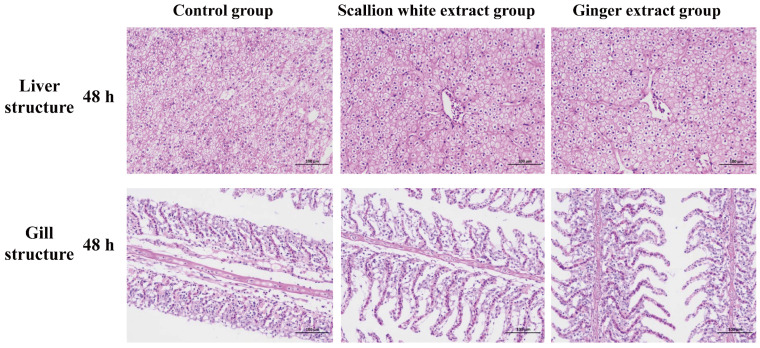
Changes in gill and liver structure of fish during transportation for 48 h. Gill and liver structures with a scale bar of 100 μm.

As shown in Figure 2, after 48 h of transportation, the liver tissue in the control group exhibited marked damage, characterized by disorganized hepatocyte arrangement, indistinct cellular boundaries, and pronounced vacuolation of hepatocytes. Regarding gill structure, the control group exhibited severe damage, characterized by extensive dissociation, disorganization, and rupture, along with marked vacuolation of chloride cells. In contrast, the scallion white extract group showed relatively orderly secondary lamellae and more intact epithelial cell structures, with alleviated damage. The ginger extract group demonstrated clearer gill architecture with well-arranged lamellae, showing more pronounced improvement in tissue integrity.

### 3.4. Changes in Blood Redox Indicators of Fish During Transportation

**Figure 3:**
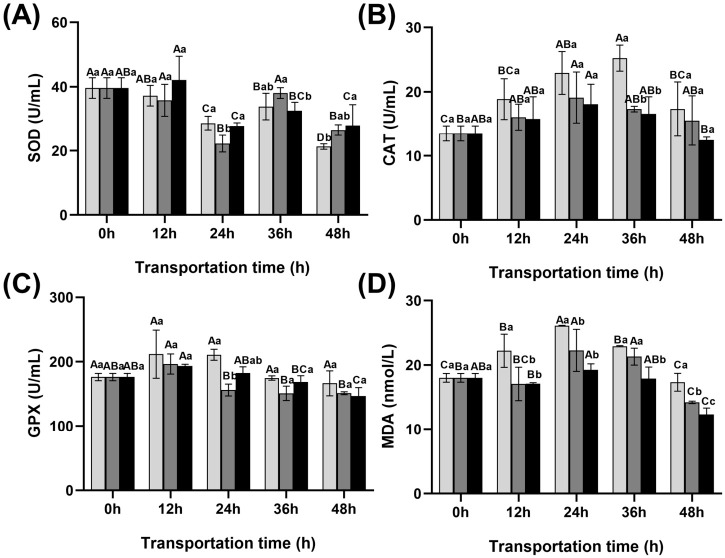
Changes in redox parameters and gene expression in blood of fish during transportation. (**A**) SOD, (**B**) CAT, (**C**) GPX, (**D**) MDA. 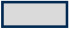 Control group 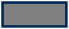 Scallion white group 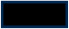 Ginger extract group. SOD: superoxide dismutase, CAT: catalase, GPX: glutathione peroxidase, MDA: malondialdehyde. Different uppercase letters indicate significant differences within the group at different time points (*p* < 0.05), while different lowercase letters indicate significant differences between groups at the same time point (*p* < 0.05).

As shown in Figure 3, SOD activity displayed dynamic fluctuations during transport, and at 48 h, it was significantly elevated in the scallion white and ginger extract groups relative to the control. After 48 h of transport, the SOD activity in the control group decreased to 21.33 U/mL, while both scallion white and ginger extract groups showed significantly higher levels compared with the control group (*p* < 0.05), reaching 26.54 U/mL and 32.13 U/mL, respectively. In addition, the activities of CAT and GPX, along with the MDA content, displayed an initial elevation followed by a gradual reduction as the transportation period was prolonged. Moreover, the scallion white and ginger extract groups exhibited reduced MDA levels compared to the control group, with the difference being most pronounced at 48 h (*p* < 0.05), reaching 14.20 and 12.33 nmol/L, respectively.

### 3.5. Changes in the Expression Levels of caspase 3, hsp70, hsp90, and il-6 in the Blood of Fish During Transportation

**Figure 4:**
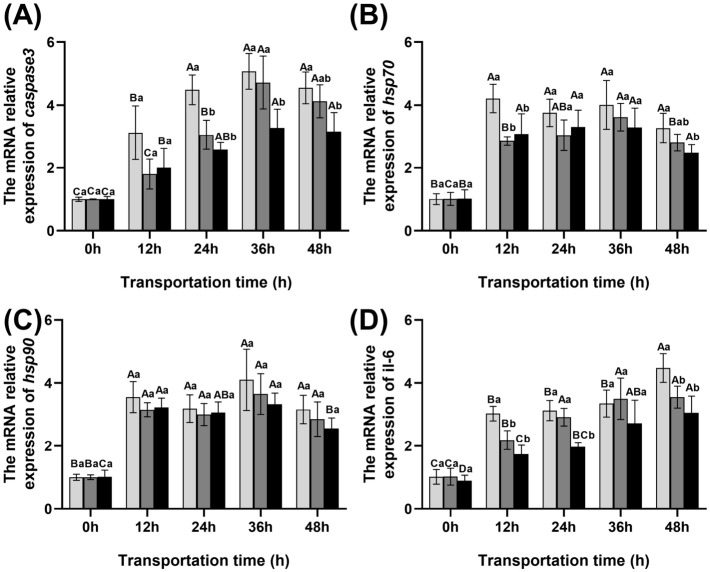
Changes in redox parameters and gene expression in blood of fish during transportation. (**A**) *caspase 3*, (**B**) *hsp 70*, (**C**) *hsp 90*, (**D**) *il-6*. 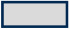 Control group 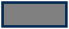 Scallion white group 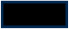 Ginger extract group. Different uppercase letters indicate significant differences within the group at different time points (*p* < 0.05), while different lowercase letters indicate significant differences between groups at the same time point (*p* < 0.05).

The changes in gene expression in the blood of fish during transport are shown in Figure 4. Between 0 and 12 h, the transcription levels of *hsp70* and *hsp90* in crucian carp were significantly upregulated (*p* < 0.05). Throughout the transport period, the transcription levels of *hsp70* and *hsp90* in the control group were higher than those in the scallion white and ginger extract groups, with the scallion white group showing higher levels than the ginger extract group. The relative expression of *caspase 3* in the control group increased progressively from 0 to 36 h, peaking at 5.07. Although expression decreased at 48 h, it remained elevated at 4.55—significantly higher than that in the scallion white (4.13) and ginger extract groups (3.16). The transcription level of *il-6* increased significantly with transport time (*p* < 0.05), reaching its highest at 48 h in all three groups, with relative expression values of 4.47, 3.55, and 3.05 in the control, scallion white, and ginger extract groups, respectively. Overall, between 12 and 48 h, the transcription levels of *il-6* and *caspase 3* in the control group were significantly higher than those in the scallion white and ginger extract groups (*p* < 0.05), with the ginger extract group consistently showing the lowest levels.

### 3.6. Changes in Muscle Quality of Crucian Carp During Transportation

#### 3.6.1. Color

As shown in Table 1, the L* and *W* values of both the control and ginger extract groups increased with prolonged transportation time, while no significant changes were observed in the scallion white extract group. The a* value of the control group reached its maximum at 12 h, reflecting the highest degree of redness at this time point. In contrast, the a* values of the scallion white and ginger extract groups remained relatively stable throughout the transportation period. Additionally, the b* values of the control and scallion white extract groups showed an increasing trend, whereas the ginger extract group exhibited no significant variation.

#### 3.6.2. Shear Force

**Figure 5:**
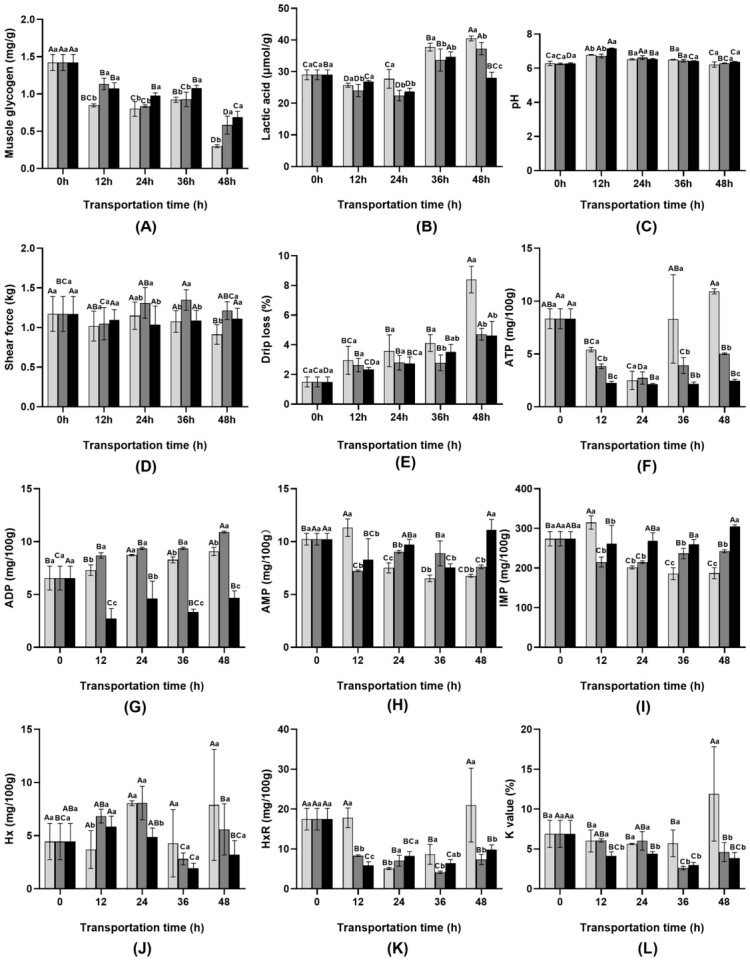
Changes in the muscle quality of fish during transportation. (**A**) Shear force, (**B**) drip loss, (**C**) muscle glycogen, (**D**) lactic acid, (**E**) pH, (**F**) ATP, (**G**) ADP, (**H**) AMP, (**I**) IMP, (**J**) Hx, (**K**) HxR, (**L**) K. 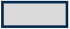 Control group. 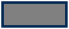 Scallion white group. 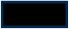 Ginger extract group. ATP: adenosine triphosphate, ADP: adenosine diphosphate, AMP: adenosine monophosphate, IMP: inosine monophosphate, Hx: hypoxanthine, HxR: inosine, K: freshness value. Different uppercase letters indicate significant differences within the group at different time points (*p* < 0.05), while different lowercase letters indicate significant differences between groups at the same time point (*p* < 0.05).

From Figure 5, it can be seen that the muscle shear force of the control group and scallion white extract shows a trend of first decreasing, then increasing, and then decreasing with the prolongation of transportation time; The muscle shear force of the ginger group first decreases and then remains stable with the extension of transportation time. At 12 h, the shear force of fish meat significantly decreased (*p* < 0.05). At 12–36 h, the shear force of the control group showed a fluctuating trend, while the scallion white extract group and ginger group showed a relatively stable or slowly increasing trend. At 48 h of transportation, the shear force of the control group was significantly lower than that of the scallion group and ginger group (*p* < 0.05).

#### 3.6.3. Drip Loss

The drip loss of muscles increases with the prolongation of transportation time. Within 0–36 h, the drip loss of the control group, scallion group, and ginger group was relatively low, fluctuating within the range of 1.49% to 4.12%. However, after 48 h of transportation, the drip loss of the control group increased sharply, reaching 8.4%, significantly higher than that of the scallion white extract group (4.71%) and the ginger group (4.63%) (*p* < 0.05).

#### 3.6.4. Muscle Glycogen, Lactic Acid and pH

In the control group, with the extension of transportation time from 0 h to 48 h, the muscle glycogen content significantly declined from 1.42 mg/g to 0.30 mg/g, whereas the lactic acid content markedly increased from 29.00 μmol/g to 40.51 μmol/g, indicating enhanced glycolytic metabolism under prolonged transport stress (Figure 5). With the extension of transportation time, the ginger extract group exhibited a slower decline in muscle glycogen content and a less pronounced increase in lactic acid levels compared with the control group. The muscle pH value increased initially and then decreased with the prolongation of transportation time (Figure 5).

#### 3.6.5. ATP and Its Metabolites Content

As shown in Figure 5, the ATP content in muscle shows a trend of first decreasing and then increasing with the prolongation of transport time. With the prolongation of transportation time, the nucleotide contents showed dynamic changes. The AMP and IMP contents in the control group first increased and then decreased, while the IMP levels in the scallion white and ginger extract groups first decreased and then increased. HxR exhibited a trend of first decreasing and then increasing, whereas Hx in the control group increased from 12 to 24 h (*p* < 0.05). AMP and IMP, as umami nucleotides, can mask the bitterness of Hx/HxR; however, prolonged stress accelerates IMP degradation to Hx, leading to flavor deterioration. After 48 h, the control group had significantly lower AMP and IMP but higher Hx and HxR contents than the extract groups (*p* < 0.05), indicating that scallion white extract and ginger extracts effectively inhibited the degradation of umami substances and the formation of bitter compounds. The K value first decreased and then increased during transportation, reaching the lowest at 36 h (5.72%, 2.61%, and 2.97% for the control, scallion white extract, and ginger groups, respectively) and rising sharply at 48 h (11.90%, 4.61%, and 3.87%; *p* < 0.05).

### 3.7. Changes in Muscle Apoptotic Gene Expression During Transportation

**Figure 6:**
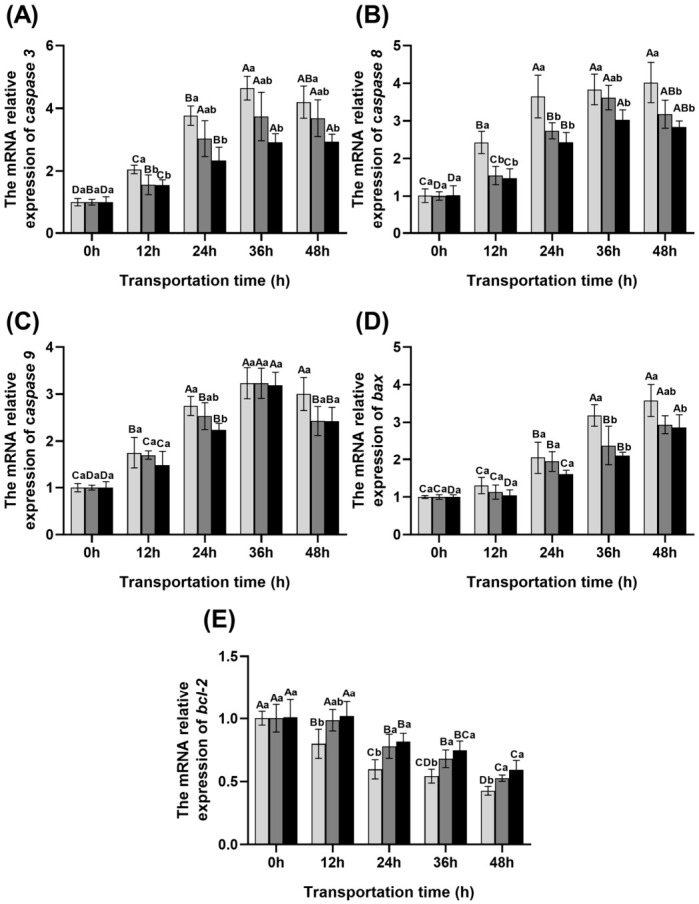
Changes in the gene expression levels of *caspase 3* (**A**), *caspase 8* (**B**), *caspase 9* (**C**), *bax* (**D**), and *bcl-2* (**E**) in the blood of fish during transportation. 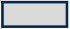 Control group. 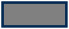 Scallion white group. 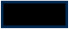 Ginger extract group. Different uppercase letters indicate significant differences within the group at different time points (*p* < 0.05), while different lowercase letters indicate significant differences between groups at the same time point (*p* < 0.05).

As shown in Figure 6, the transcription levels of *caspase 3*, *caspase 8*, *caspase 9*, and *bax* all exhibited an upward trend with prolonged transport time. with transportation time, whereas *bcl-2* decreased. However, in the scallion white extract and ginger extract groups, *caspase* and *bax* expression was significantly lower, and *bcl-2* expression was significantly higher than in the control group (*p* < 0.05), with ginger exhibiting the strongest inhibitory effect on *caspase 3* and *caspase 8* gene expression.

### 3.8. Changes in Muscle Structure of Fish During Transportation

**Figure 7:**
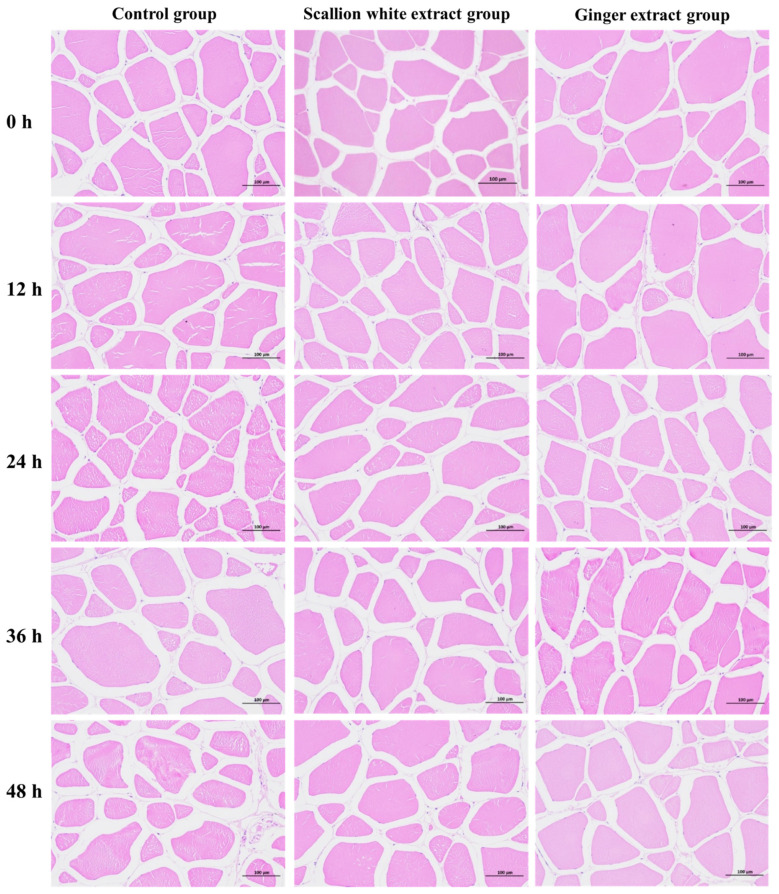
Changes in cellular structure of fish during transportation. Cellular structure with a scale bar of 100 μm.

As shown in Figure 7, with prolonged transportation, the gaps between muscle cells gradually widened, muscle fiber breakage intensified, and the overall breakage rate increased. Previous studies have demonstrated that transport stress can damage muscle cell structure, thereby affecting muscle quality. For instance, Peng et al. [12] reported that during transportation, Blunt snout bream (*Megalobrama amblycephala*) exhibited enlarged muscle fiber gaps and increased fiber breakage.

During the initial 0–24 h, only slight enlargement of muscle cell gaps and local fiber rupture were observed, with no significant differences among the control, scallion, and ginger groups. At 36 h, muscle fiber gaps in the control group increased significantly, and the previously tight structure was disrupted. By 48 h, fiber gaps were further enlarged, and severe cracking occurred in some regions. In contrast, the scallion and ginger groups maintained smaller cell gaps and tighter fiber arrangements at 36–48 h.

## 4. Discussion

### 4.1. The Supplementation of Natural Antioxidants Effectively Improved Water Quality Parameters During Transportation

Before sealing, the air inside the fish bags was largely removed, resulting in a low initial DO concentration in the water (Figure S1). The bags were then sealed and filled with oxygen. Under high-pressure conditions, the dissolution rate of oxygen exceeded the oxygen consumption rate of fish, leading to a gradual increase in DO concentration within 0–24 h [13]. Furthermore, as the fish continuously consumed oxygen and the bag provided a closed environment without external oxygen supply, the DO gradually decreased. During prolonged transportation, the fish exhibited heightened activity, increased respiration, and enhanced metabolism, leading to substantial CO_2_ release through the gills and fecal excretion, which dissolved in the water to form carbonic acid and ammonia nitrogen [14]. The accumulation of ammonia nitrogen subsequently contributed to increases in nitrite and total dissolved solids (TDS). It is worth noting that the supplement of ginger extract mitigated ion loss by protecting gill function and reducing oxidative damage [15]. Nitrite levels increased during 12–48 h, with the ginger group showing higher concentrations than control and scallion white extract groups, possibly due to inhibition of nitrifying bacteria activity by gingerol [16]. Ginger and scallion white extracts effectively improved water quality parameters during transportation.

### 4.2. The Supplementation of Natural Antioxidants Effectively Alleviated Stress Responses in Crucian Carp During Transportation

Compared with the control group, the supplementation of ginger and scallion white extracts resulted in overall lower antioxidant enzyme activities and MDA levels, indicating that both natural extracts alleviated oxidative stress during transportation. In the early stage (0–12 h), CAT, GPx, and MDA increased most markedly in the control group, whereas the changes were less pronounced in the treatment groups, suggesting that the extracts exerted protective effects from the beginning of transport. At 48 h, SOD activity significantly decreased in the control group (*p* < 0.05), while it remained relatively higher in both the ginger and scallion white extracts groups, indicating that these extracts helped maintain antioxidant defense capacity. Overall, both extracts contributed to stabilizing the antioxidant system, with ginger showing a comparatively stronger effect. Furthermore, supplementation with ginger and scallion white extracts enhanced antioxidant defense capacity, which was associated with reduced cortisol production, lower energy substrate consumption, and alleviated liver and kidney damage. Specifically, the levels of cortisol, CHOL, LDH, GLU, UREA, CREA, and AST were all lower than those in the control group, and the liver and kidney structures remained more intact and morphologically normal.

Compared with the control group, supplementation of ginger and scallion white extracts into the transport water significantly reduced the transcription levels of *caspase3*, *hsp70*, and *il-6* in the muscle of crucian carp. Hsp70, a typical molecular chaperone, is an important marker for maintaining protein homeostasis under stress conditions, and its elevated expression generally reflects increased oxidative damage and protein misfolding stress in the organism. IL-6 is a key pro-inflammatory cytokine involved in inflammatory cascades and immune activation, whereas Caspase-3 is the central executioner in the apoptotic pathway, and its upregulation often indicates enhanced apoptosis and aggravated tissue damage. Therefore, the decreased expression of these genes suggests that the addition of ginger and scallion white extracts effectively alleviated oxidative stress, inflammatory response, and apoptosis in crucian carp during transportation.

### 4.3. Mechanism of Ginger and Scallion White Extracts in Improving Fish Muscle Quality

**Figure 8:**
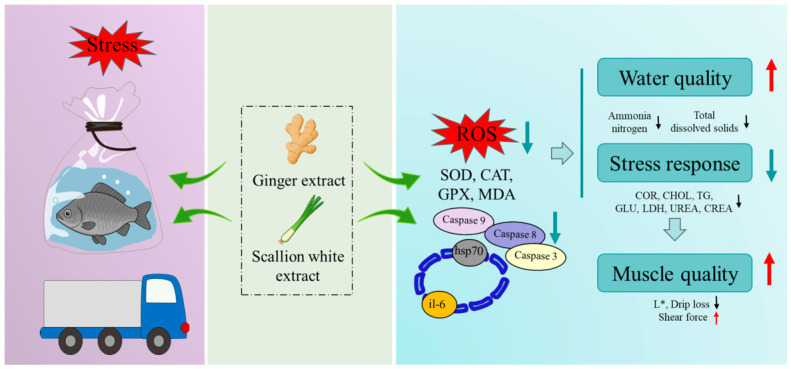
Ginger and scallion extracts alleviate transport-induced stress and improve muscle quality in fish. SOD: superoxide dismutase, CAT: catalase, GPX: glutathione peroxidase, MDA: malondialdehyde, L*: lightness, ↑: upregulation, ↓: downregulation.

Scallion white and ginger extracts help alleviate systemic stress responses by enhancing antioxidant defense and stabilizing metabolic homeostasis, while simultaneously improving water quality through their natural antimicrobial and bioactive compounds, thereby reducing environmental stressors during transportation. The detailed mechanism of action is illustrated in Figure 8.

At the molecular level, the extracts exhibit strong antioxidant activity by scavenging ROS, enhancing cellular redox balance, and mitigating oxidative damage induced by transport stress. In parallel, they modulate multiple stress-related pathways, including heat shock protein response, inflammatory response, and apoptosis signaling. After the addition of the extracts, the expression levels of heat shock proteins *hsp70* and *hsp90* were significantly lower than those in the control group. These proteins are known to play crucial roles in stress defense, hormone signal transduction, cell cycle regulation, cell proliferation and differentiation, and apoptosis [17]. Moreover, the expression level of the inflammation-related gene *il-6* was also reduced, indicating an attenuation of inflammatory response [18]. In addition, the extracts upregulated the anti-apoptotic gene *bcl-2* and downregulated pro-apoptotic genes (*caspase 3*, *caspase 8*, *caspase 9*, and *bax*), thereby protecting muscle cells from apoptosis and structural destruction [19].

In terms of cellular structure, heat shock proteins (HSPs) can induce protein denaturation under stress conditions, leading to increased cell membrane permeability and expanded intercellular spaces, thereby disrupting the integrity of muscle tissue [20]. Excessive apoptosis further exacerbates these structural changes by causing tissue loosening and disorganized cellular arrangement. For instance, in fish muscle, myofiber apoptosis results in fiber breakage, myofibril disassembly, and enlarged intercellular spaces [21,22]. In addition, inflammatory responses, mediated by pro-inflammatory cytokines such as *il-6*, promote the generation of ROS and nitric oxide (NO), triggering oxidative damage and mitochondrial dysfunction. These inflammatory processes collectively increase membrane permeability, induce myofiber breakage, expand intercellular spaces, and further compromise the structural integrity of muscle tissue [23]. The supplementation of scallion white and ginger extracts effectively suppresses stress-related cellular responses, thereby preserving membrane integrity and mitigating muscle structural damage. Muscle cellular damage disrupts myofiber integrity, reducing shear force and causing water to shift from the intracellular to extracellular space, which increases drip loss and free water content on the muscle surface, thereby elevating L* values [1].

Furthermore, scallion and ginger extracts reduced ATP consumption, leading to more stable ATP levels than in the control group [24]. The Hx contents in the extract groups were consistently lower than in the control, possibly because active components inhibited AMP deaminase or scavenged ROS, reducing oxidative interference with nucleotide metabolism. AMP and IMP, as umami nucleotides, can mask the bitterness of Hx/HxR; however, prolonged stress accelerates IMP degradation to Hx, leading to flavor deterioration. After 48 h, the control group had significantly lower AMP and IMP but higher Hx and HxR contents than the extract groups (*p* < 0.05), indicating that scallion white extract and ginger extracts effectively inhibited the degradation of umami substances and the formation of bitter compounds. Overall, the control group had the highest K value and the lowest freshness, whereas the scallion white extract and ginger extract groups maintained lower K values, suggesting better preservation of IMP/Hx balance and delayed freshness loss by stabilizing nucleotide metabolism [24]. Supplementation with scallion white and ginger extracts effectively preserves muscle structure and improves overall quality, with ginger extract exhibiting stronger protective effects than scallion extract.

## 5. Conclusions

This study shows that scallion white and ginger extracts alleviate transport-induced stress in crucian carp by improving water quality, regulating stress-related indicators, and preserving muscle quality. Ginger extract exhibited stronger effects than scallion white extract, particularly in reducing oxidative stress- and apoptosis-related responses. These findings support the potential application of plant-derived extracts as natural additives to improve fish welfare and product quality during live fish transportation.

## Figures and Tables

**Table 1:** Changes in muscle color of fish during transportation.

Transportation Time/h	Group	L*	a*	b*	*W*
0	Control group	46.83 ± 2.20 ^Ca^	0.41 ± 0.26 ^Da^	1.07 ± 0.47 ^Ca^	46.82 ± 2.20 ^Ca^
	Scallion white extract group	48.32 ± 2.32 ^Aa^	0.55 ± 0.19 ^Aa^	1.35 ± 0.68 ^Ca^	48.3 ± 2.31 ^Aa^
	Ginger extract group	47.94 ± 0.47 ^Ba^	0.43 ± 0.45 ^Aa^	1.03 ± 0.32 ^Aa^	47.92 ± 0.46 ^Ba^
12	Control group	51.23 ± 2.29 ^Ba^	1.71 ± 0.16 ^Aa^	4.73 ± 0.55 ^Ba^	50.94 ± 2.24 ^Ba^
	Scallion white extract group	49.52 ± 3.82 ^Aab^	0.48 ± 0.26 ^Ab^	1.68 ± 0.83 ^BCb^	49.48 ± 3.80 ^Aab^
	Ginger extract group	45.93 ± 1.35 ^Bb^	0.83 ± 0.47 ^Aab^	1.21 ± 0.75 ^Ab^	45.9 ± 1.35 ^Bb^
24	Control group	49.86 ± 1.43 ^Ba^	0.76 ± 0.20 ^Ca^	4.41 ± 1.64 ^Ba^	49.63 ± 1.39 ^Ba^
	Scallion white extract group	47.78 ± 1.93 ^Aab^	0.36 ± 0.12 ^Bb^	4.98 ± 0.69 ^Aa^	47.53 ± 1.92 ^Aab^
	Ginger extract group	46.19 ± 1.91 ^Bb^	0.99 ± 0.45 ^Aa^	1.71 ± 0.67 ^Ab^	46.15 ± 1.92 ^Bb^
36	Control group	55.07 ± 1.84 ^Aa^	1.26 ± 0.15 ^Ba^	6.7 ± 1.70 ^Aa^	54.53 ± 1.68 ^Aa^
	Scallion white extract group	48.83 ± 2.40 ^Ab^	0.65 ± 0.47 ^Aa^	3.23 ± 1.81 ^ABb^	48.7 ± 2.29 ^Ab^
	Ginger extract group	50.57 ± 2.22 ^Ab^	1.05 ± 0.65 ^Aa^	1.44 ± 0.75 ^Ab^	50.53 ± 2.25 ^Ab^
48	Control group	55.07 ± 1.59 ^Aa^	1.15 ± 0.19 ^Ba^	5.26 ± 0.52 ^ABa^	54.74 ± 1.54 ^Aa^
	Scallion white extract group	49.49 ± 1.18 ^Ab^	0.61 ± 0.32 ^Ab^	3.37 ± 1.30 ^ABb^	49.36 ± 1.25 ^Ab^
	Ginger extract group	50.35 ± 1.31 ^Ab^	0.55 ± 0.16 ^Ab^	1.26 ± 0.18 ^Ac^	50.33 ± 1.31 ^Ab^

Note: L*: brightness, a*: red/green, b*: yellow/blue, W: whiteness. Different uppercase letters indicate significant differences within the group at different time points (*p* < 0.05), while different lowercase letters indicate significant differences between groups at the same time point (*p* < 0.05).

## Data Availability

The original contributions presented in this study are included in the article/Supplementary Material. Further inquiries can be directed to the corresponding authors.

## Supplementary Materials

The following supporting information can be downloaded at: https://www.mdpi.com/article/10.3390/foods15101645/s1. Figure S1: Changes in water quality indicators during different transport durations. Table S1: Primers used for quantitative PCR in serum. Table S2: Primers used for quantitative PCR in muscle.
