# Supplementary material for: Scallion White and Ginger Extracts Alleviate Stress-Induced Muscle Quality Deterioration in Crucian Carp During Transportation
Source: Foods. 2026 May 8;15(10):1645. doi: 10.3390/foods15101645 (PMC13206114; doi:10.3390/foods15101645)
Supplement: Supplementary file 1 [file foods-15-01645-s001.zip › foods-4109058-supplementary.pdf]

Figure S1. Changes in water quality indicators during different transport durations

Note:(A) pH; (B) dissolved oxygen; (C) ammonia nitrogen concentration; (D) TDS; and (E) nitrite concentration. Different uppercase letters indicate significant differences within the group at different time points ($p < 0.05$), while different lowercase letters indicate significant differences between groups at the same time point ($p < 0.05$).

Control group Scallion white group Ginger extract group. TDS: total dissolved solids.

Figure S1.

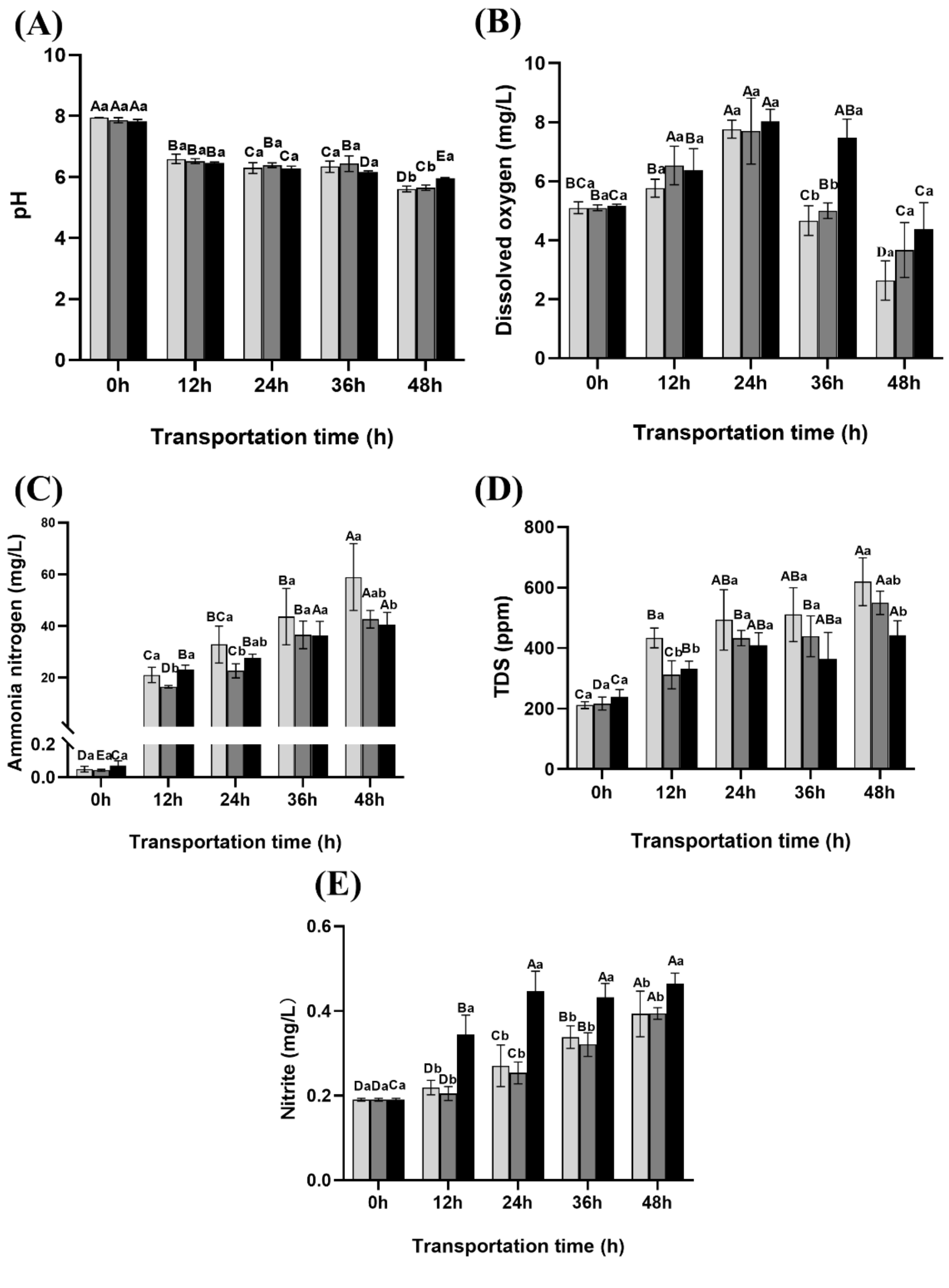

(A)
pH
Transportation time (h)
0h
12h
24h
36h
48h
(B)
Dissolved oxygen (mg/L)
Transportation time (h)
(C)
Ammonia nitrogen (mg/L)
Transportation time (h)
(D)
TDS (ppm)
Transportation time (h)
(E)
Nitrite (mg/L)
Transportation time (h)

Table S1 Primers used for quantitative PCR in serum

Target gene	Primers	Primer sequence (5’→3’)	Note
hsp 70	*hsp 70-S*	ACACGACTGGTCCATTTCTGC	
	hsp 70-A	ACTTCGTTGTCTCTCCGGTC	
hsp 90	*hsp 90-S*	ATGAGGACAAGGACAAACCGAA	
	hsp 90-A	GTCTTGTAGAAGGCTTTGTATCGTC	
caspase 3	*caspase3-S*	ACAGGCATGAACCAACGGAA	
	caspase3-A	ACACACTAACGAAGCACAACG	
il-6	*il-6-S*	GACGGCTGTCTGTCCAGAAACT	
	il-6-A	GATGTCGTTGACCAGGGTTGAG	
gapdh	*gapdh -S*	AGGCATTCTGGGATACACGGAG	reference genes
	gapdh -A	GATGGGAGAACGGTGGGTCA	

Note: “S” at the end of the primer name refers to the Sense primer, and “A” refers to the Anti-sense primer.

Table S2. Primers used for quantitative PCR in muscle

Target gene	Primers	Primer sequence (5'→3')	Note
Caspase-3	*caspase3-S*	ACAGGCATGAACCAACGGAA	
	caspase3-A	ACACACTAACGAAGCACAACG	
Caspase-8	*caspase8-S*	TACGACTGAACGAGCAAGCA	
	caspase8-A	ATGCGTCACGTTGTAGCAGA	
Caspase-9	*caspase9-S*	AACAAGACGTGACCAAGCCAG	
	caspase9-A	GCGAAGGCTGTATGGGGACA	
Bcl-2	*bcl2-S*	CTGATGCCTTTTTGGCCGTTG	
	bcl2-A	CGACACTAGGCTCTTGCGA	
Bax	*bax-S*	CTTTGCGTGTCGGCTTGTC	
	bax-A	CTCCCATCCACCCTGTTCC	
gapdh	*gapdh -S*	AGGCATTCTGGGATACACGGAG	reference genes
	gapdh -A	GATGGGAGAACGGTGGGTCA	

Note: "S" at the end of the primer name refers to the Sense primer, and "A" refers to the Anti-sense primer.
